# Riboflavin-Induced Disease Resistance Requires the Mitogen-Activated Protein Kinases 3 and 6 in *Arabidopsis thaliana*

**DOI:** 10.1371/journal.pone.0153175

**Published:** 2016-04-07

**Authors:** Shengjun Nie, Huilian Xu

**Affiliations:** International Nature Farming Research Center, Hata 5632, Matsumoto-city, Nagano 390–1401, Japan; Hainan University, CHINA

## Abstract

As a resistance elicitor, riboflavin (vitamin B_2_) protects plants against a wide range of pathogens. At molecular biological levels, it is important to elucidate the signaling pathways underlying the disease resistance induced by riboflavin. Here, riboflavin was tested to induce resistance against virulent *Pseudomonas syringae* pv. *Tomato* DC3000 (*Pst* DC3000) in Arabidopsis. Results showed that riboflavin induced disease resistance based on MAPK-dependent priming for the expression of *PR1* gene. Riboflavin induced transient expression of *PR1* gene. However, following *Pst* DC3000 inoculation, riboflavin potentiated stronger *PR1* gene transcription. Further was suggested that the transcript levels of mitogen-activated protein kinases, MPK3 and MPK6, were primed under riboflavin. Upon infection by *Pst* DC3000, these two enzymes were more strongly activated. The elevated activation of both MPK3 and MPK6 was responsible for enhanced defense gene expression and resistance after riboflavin treatment. Moreover, riboflavin significantly reduced the transcript levels of MPK3 and MPK6 by application of AsA and BAPTA, an H_2_O_2_ scavenger and a calcium (Ca^2+^) scavenger, respectively. In conclusion, MPK3 and MPK6 were responsible for riboflavin-induced resistance, and played an important role in H_2_O_2_- and Ca^2+^-related signaling pathways, and this study could provide a new insight into the mechanistic study of riboflavin-induced defense responses.

## Introduction

To survive in unfavorable environments, plants have evolved sophisticated defense strategies to induce their immune system against pathogens and protect themselves from being infected. Upon appropriate biotic or abiotic stimulation, plants can develop enhanced capacity to express pathogen-induced defense response [1, 2]. The phenomenon has been known as priming, and this priming can result in a faster and/or stronger induction of defense mechanisms when subsequently challenged by pathogens [3, 4]. For example, inoculation with *Pseudomonas syringae* pv tomato (*Pst*) strain DC3000 expressing avrRpt2 gene was shown to enhance defense response that were manifested following *Pst* DC3000 infection [5]. Following infection by the pathogen, plants can develop enhanced resistance when subsequently challenged by other pathogens attack. The type of induced resistance is regarded as systemic acquired resistance (SAR) that is mediated by endogenous accumulation of the plant hormone salicylic acid (SA) [6, 7]. In addition, the induced resistance is associated with the accumulation of pathogenesis-related (PR) protein, and in fact, the so-called priming of defense in cells has been known as a component of induced resistance response in plants [5, 8].

In addition to pathogens attack, exogenous application of specific compounds and synthetic chemicals, can also induce this form of resistance in plants [3]. For example, the non-protein amino acid β-amino-butyric acid (BABA) induced resistance against the bacterial pathogen *Pst* DC3000 and the fungal pathogen *B*. *cinerea* [9, 10], and BABA-induced resistance against B. cinerea is based on ABA-dependent priming for callose accumulation [11]. Thiamine is a B-complex that is produced in plants and microbes. Thiamine functions as a plant defense activator, and confers SAR through priming which requires hydrogen peroxide and intact signaling protein NON EXPRESSOR OF PR1 (NPR1) [12, 13]. Thus, plants primed by treatments that induce resistance show a faster and stronger activation of defense response after pathogens attack.

Riboflavin (vitamin B_2_), as a coenzyme in many physiological processes, is produced by plants and microbes [14–16]. Riboflavin is an essential nutrient for humans. Riboflavin deficiency causes skin and mucosal disorders, including cheilitis and anemia [17]. Riboflavin exists in three forms: free riboflavin and two cofactor forms, flavin mononucleotide (FMN) and flavin adenine dinucleotide (FAD). These forms act as coenzymes in physiological processes, including light sensing, bioluminescence and DNA repair [18]. In addition to riboflavin’s well-known nutritional value and as an enzyme cofactor, recent studies have demonstrated a novel function of riboflavin in disease resistance [19, 20]. Treatment with riboflavin protects tobacco and Arabidopsis from fungal and bacterial infections without inhibiting pathogens growth. Riboflavin activates PR genes in Arabidopsis which is dependent on the *NPR1* gene and induces SAR to pathogens, suggesting that riboflavin initiates resistance signal transduction. Although priming induced by riboflavin has been known as a component of resistance responses in plants [19, 20], very little is known about the molecular mechanisms of priming defense by riboflavin.

Recently, it has been suggested that priming is associated with enhanced accumulation of cellular signaling proteins when subsequently challenged by stress [3, 9]. Alteration to the phosphorylation state of these signaling proteins plays an important role in signal transduction. Mitogen-activated protein kinase (MAPK) cascades are an important part of the signaling machinery that transduces extracellular stimuli into intracellular responses in all eukaryotic cells [21–26]. This cascade generally consists of three functional kinases, a MAP kinase kinase kinase (MAPKKK), a MAP kinase kinase (MAPKK), and a MAPK. In Arabidopsis, 20 MAPKs, 10 MAPKKs and 60 MAPKKKs have been identified [21, 27, 28]. Furthermore, MAPKs have been implicated in regulating innate immunity and adverse stress responses in plants and animals [29–31]. Because MAPK cascades play essential roles in cellular signal amplification, MAPKs, MAPKKs and MAPKKKs are excellent candidates for signaling enzymes mediating priming. Recent investigations have verified a MAPK cascade, extending from MEKK1 through MEK4/5 to MPK3/6 in response to the microbe-associated molecular pattern flagellin, or its conserved N-terminal 22-amino-acid epitope flg22 [21]. MPK3 and MPK6 can be activated by various environmental stresses and participate in Arabidopsis defense responses [32–37].

Reactive oxygen species (ROS) and calcium (Ca^2+^) are regarded as the key signaling molecules in plant cells [38, 39]. In Arabidopsis, application of hydrogen peroxide (H_2_O_2_) stimulated Ca^2+^ influx through Ca^2+^-permeable channel [38, 40, 41]. Furthermore, ROS and Ca^2+^ have been observed in numerous plant-pathogen interactions, such as hypersensitive response (HR), and these signaling messengers are up-regulated prior to the expansion of the local lesions [42–46]. Recent studies have shown that both thiamine- and riboflavin-induced resistance are dependent on H_2_O_2_ and a functional *NPR1* gene in Arabidopsis [12, 13, 19, 20], and thiamine induces priming for pathogen defense through the Ca^2+^-related signaling pathway [12].

Although the involvement of MPK3 and MPK6 in the activation of Arabidopsis defense responses is evident, a clear picture of their contribution to riboflavin-induced resistance to pathogen is still missing. In this work, the roles played by MPK3 and MPK6 in riboflavin-induced resistance to *Pst* DC3000 were investigated to see whether riboflavin increases the levels of MPK3 and MPK6 transcript and transient expression of *PR1* gene and whether or not the activities of MPK3 and MPK6 were strongly enhanced upon infection by *Pst* DC3000, with effects of riboflavin exerted through H_2_O_2_- and Ca^2+^-related signaling pathways. Examinations were made to confirm whether MPK3 and MPK6, as important signaling components, could be responsible for riboflavin-induced disease defense in Arabidopsis.

## Materials and Methods

### Plant materials and chemical treatments

Plants of Arabidopsis ecotype Col-0 and mutants lacking MAPK genes (*mpk3-1*, SALK_151594; *mpk6-2*, SALK___073907; *mpk6-3*, and SALK_127507; purchased from NASC/ABRC) were used as materials. In the *mpk3* and *mpk6* mutants (*mpk3*, *mpk6-2* and *mpk6-3*), they lack detectable transcripts of *MPK3* and *MPK6*, respectively (S1 Fig). All the plants were grown in a growth chamber with photoperiod of 16 h with a over-canopy lighting of photosynthetic photon flux of 120 μmol m^-2^ s^-1^ and 80% relative humidity at 22°C for 2- to 3 weeks. The plants were sprayed with water or 0.6 mM riboflavin in the presence of Silwet L-77 (0.015%) 4 h before pathogen Pst DC3000 was inoculated, unless stated otherwise. Here, the DC3000 strain of *Pseudomonas syringae* pv *tomato* without any other avirulent genes was lyophilized and stored at -80°C, and cultured on King’s B medium supplemented with appropriate antibiotics before inoculation. AsA, BAPTA-AM and PD98059 were purchased from Sigma-Aldrich China (Shanghai, China).

### Pathogen maintenance and pathogen challenge

*Pst* DC3000 was cultivated on the King’s B liquid medium supplemented with 75 μg mL^-1^ rifampicin at 28°C overnight and shaken until midlog growth phase (OD_600_ = 0.15) was obtained. To inoculate Arabidopsis with *Pst* DC3000, bacterial cells were retrieved from medium by centrifugation at 3,000*g* for 10 min and resuspended in 10 mM MgCl_2_, and the concentration was adjusted to 0.01 (OD_600_) in 10 mM MgCl_2_. At least 25 plants of Arabidopsis ecotype Col-0 or mutants were inoculated per treatment. Arabidopsis leaves were inoculated with the bacterial suspension of OD_600_ = 0.01 with 1-mL syringes. The inoculated plants were kept in a dew chamber for 16 h at 25°C and 100% relative humidity and then transferred to a growth chamber with a 16-h light/8-h dark regime at 25°C and 80% relative humidity. The bacterial growth was assessed by determining the CFU of 1 g FW (fresh weight) of leaves from five plants through plating appropriate dilutions on King’s B medium containing 75μg mL^-1^ rifampicin [47].

### Treatment with MAPK cascade inhibitor

The Arabidopsis leaves were pre-inoculated in a solution containing a MAPK cascade inhibitor PD98059 dissolved in DMSO for 50 min before riboflavin treatment. PD98059 was used to inhibit the activation of MAPK cascade at a final concentration of 150 μM.

### Measurements of endogenous riboflavin, FMN and FAD

The leaves were harvested at fresh weight of 100 mg and were frozen with liquid notrogen to temporarily stop enzyme activity, and then the endogenous riboflavin, FMN and FAD were extracted at the indicated time points as described by Vorwieger *et al*. [48] and Asai *et al*. [49].

### Total RNA Extraction and Real-time Quantitative RT-PCR

Total RNA was isolated from seedlings frozen in liquid nitrogen with TRIZOL reagent (Invitrogen) according to the manufacturer’s protocol. Concentration of RNA was determined by measuring OD at 260 nm. First-strand cDNA was synthesized with the SuperScript II First-Strand Synthesis System for RT-PCR (Invitrogen). Real-time quantitative PCR (qRT-PCR) analysis was performed using the LightCycler Quick System 350S (Roche Diagnostics K.K.) with SYBR Premix Ex Taq (Takara Bio, Inc.). Each PCR reaction contained 1 × SYBR Premix Ex Taq, 0.2 μM of each primer, and 2 μL of a 1:10 dilution of the cDNA in a final volume of 20 μL. The PCR programme included: the initial denaturation, at 95°C, for 30 s; PCR, of 40 cycles at 95°C, for 5 s, and then at 60°C, for 20 s with a temperature transition rate of 20°C s^-1^. In melting curve analysis, PCR reactions were denatured at 95°C, annealed at 65°C, then a monitored release of intercalator from PCR products or primer dimmers by an increase to 95°C with a temperature transition rate of 0.1°C s^-1^. Standard curves were created using PCR products by 10-fold serial dilutions. The *MPK3* and *MPK6* genes expression profiles were normalized using *actin* mRNA as an internal control. The primers for Real-time PCR are listed in S1 Table.

### GUS Staining

The transgenic PR1:GUS seedlings were grown on MS medium for about 10 days, and then transferred to liquid MS medium with or without 0.6 mM riboflavin, and 4 hours later were inoculated with *Pst* DC3000 for the indicated times. Histochemical detection of the GUS enzyme activity was analyzed as described by Gust *et al*. [50]. After staining, the seedlings were boiled in 95% ethanol for about 10 min to remove chlorophyll.

### Western blot and MAPK activity assay

Proteins were extracted from frozen leaf samples at the indicated time points after washed with sterile water as described by Liu and Zhang [34]. Protein extracts were separated on a 10% (w/v) SDS-PAGE minigel and then western blot was performed. Plant MAPKs have high homology to mammalian ERK1/2 MAPKs, and ERK1/2 antisera that recognize the dually phosphorylated (pTEpY) forms of activated MAPKs can be used to monitor plant MAPK activity [51]. Hence, endogenous kinase activity of MPK3 and MPK6 after *Pst* DC3000 inoculation was determined using phospho-P44/42 MAPK antibody (Cell Signaling Technology). Subsequently, blots were washed and incubated with an anti-rabbit horse-radish peroxidase secondary antibody. Antibodies against MPK3 and MPK6 were purchased from Sigma.

## Results

### Riboflavin induces resistance against *Pst* DC3000

**Fig 1:**
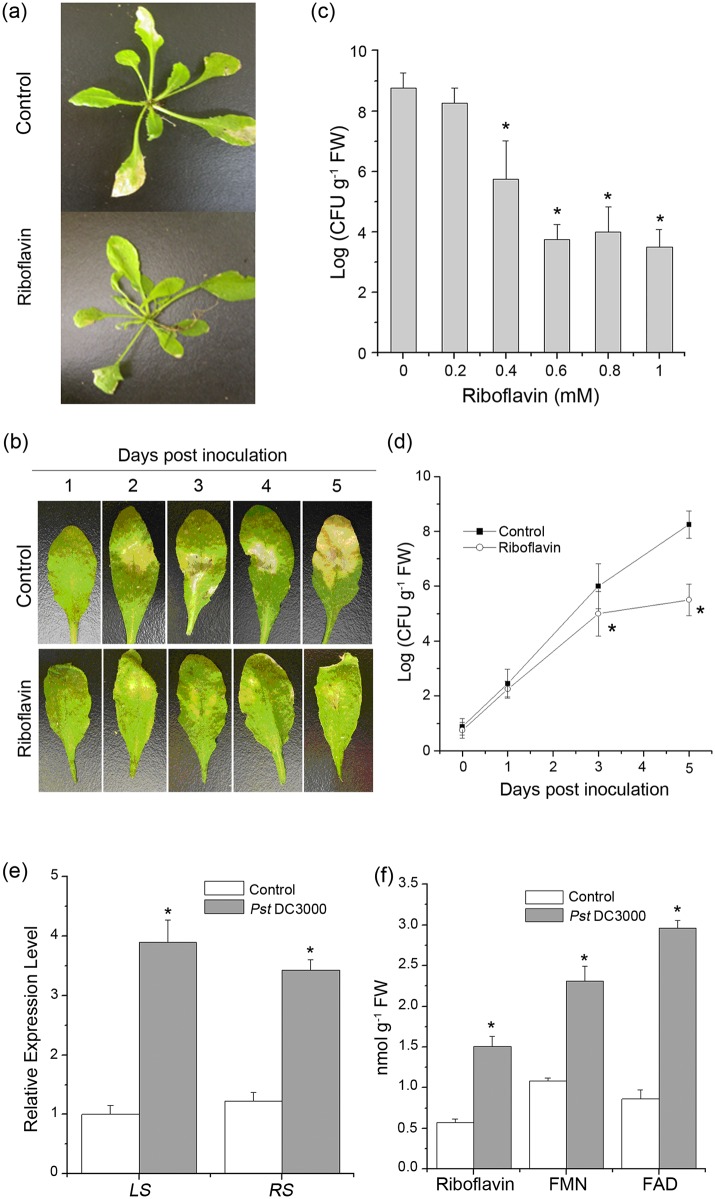
Effect of riboflavin on disease development in Arabidopsis infected with *Pst* DC3000. (**a**) 2-week-old Arabidopsis ecotype Col-0 plants were treated with either water (Control) or riboflavin (0.6 mM) and 4 hours later were inoculated with *Pst* DC3000. Infection was observed 5 days after inoculation. (**b**) Representative Arabidopsis leaves at 1, 2, 3, 4, and 5 days after infected by *Pst* DC3000. The necrotic lesions on Arabidopsis leaves infected by *Pst* DC3000 are suppressed in riboflavin-pretreated plants. (**c**) Numbers of *Pst* DC3000 in leaves of Arabidopsis treated with increasing concentrations of riboflavin (0–1 mM) prior to *Pst* DC3000 inoculation. Samples were collected 5 days after inoculation. Asterisks indicate significant differences to riboflavin-untreated samples (Student’s t-test, P <0.05). (**d**) Inhibitory effect of riboflavin on *Pst* DC3000 growth in Arabidopsis. The Arabidopsis leaves were inoculated with *Pst* DC3000 4 hours after riboflavin treatment. Samples were collected during 5 days after inoculation. Asterisks indicate significant differences to control (Student’s t-test, P <0.05). (**e**) Changes in biosynthesis genes of riboflavin, including lumizine synthase (LS) and riboflavin synthase (RS) genes, in response to Pst DC3000 in Arabidopsis. (**f**) Quantification of endogenous riboflavin, flavin mononucleotide (FMN) and flavin adenine dinucleotide (FAD) in Arabidopsis inoculation with Pst DC3000. Data are means ± SD of three replicates.

To evaluate whether riboflavin-induced plant defense is effective against virulent *Pseudomonas syringae* pv *tomato* strain DC3000 (*Pst* DC3000), 2-week-old Arabidopsis ecotype Col-0 plants were treated with either water or riboflavin, and subsequently challenged with *Pst* DC3000. Most leaves without riboflavin treatment (Control) exhibited light yellow 2 days after challenge inoculation, and finally wilted and died 5 days after inoculation. In contrast, pre-treated with 0.6 mM riboflavin, plants showed no visible symptoms 2 days after inoculation. Following 3 to 5 days, minute spots were observed on the same leaves (Fig 1a and 1b), but no further symptoms of disease were seen thereafter. In addition, compared with water-treated control plants, treatment with 0.6 mM riboflavin induced a statistically significant reduction in bacterial growth from 3 days after challenge inoculation (Fig 1d).

To determine the effects of riboflavin on the growth of *Pst* DC3000, Arabidopsis plants were treated with Riboflavin to concentrations ranging from 0 to 1 mM. Riboflavin did not result in any remarkable alterations in the plants. The bacterial number was reduced significantly with 0.4 mM and further by 0.6 mM, and the effect of further higher concentrations (0.8–1 mM) of riboflavin on the bacterial growth was similar to that of 0.6 mM (Fig 1c), indicating that 0.6 mM is sufficient for subsequent experiments in riboflavin-induced resistance against *Pst* DC3000.

### Levels of endogenous riboflavin, and its derivates, FMN and FAD in response to *Pst* DC3000

To investigate whether the endogenous riboflavin biosynthesis was influenced by *Pst* DC3000 infection, we analyzed the expression of genes responsible for the biosynthesis of the lumizine synthase (LS) and riboflavin synthase (RS), which catalyze the last two step responses in the biosynthesis of riboflavin in organisms [48, 52]. A scheme showing the different steps in riboflavin and its derivatives, flavin mononucleotide (FMN) and flavin adenine dinucleotide (FAD) biosynthesis is given in S2 Fig Results showed that the expression of genes *LS* and *RS* were up-regulated in different degrees after challenged with *Pst* DC3000 (Fig 1e). Correlated with the up-regulation of the genes, the changes in riboflavin and its derivatives, FMN and FAD, were also elevated (Fig 1f), suggesting that plants increased riboflavin biosynthesis upon *Pst* DC3000 infection.

### Riboflavin-induced resistance against *Pst* DC3000 is based on MAPK-dependent priming for the expression of *PR1*

**Fig 2:**
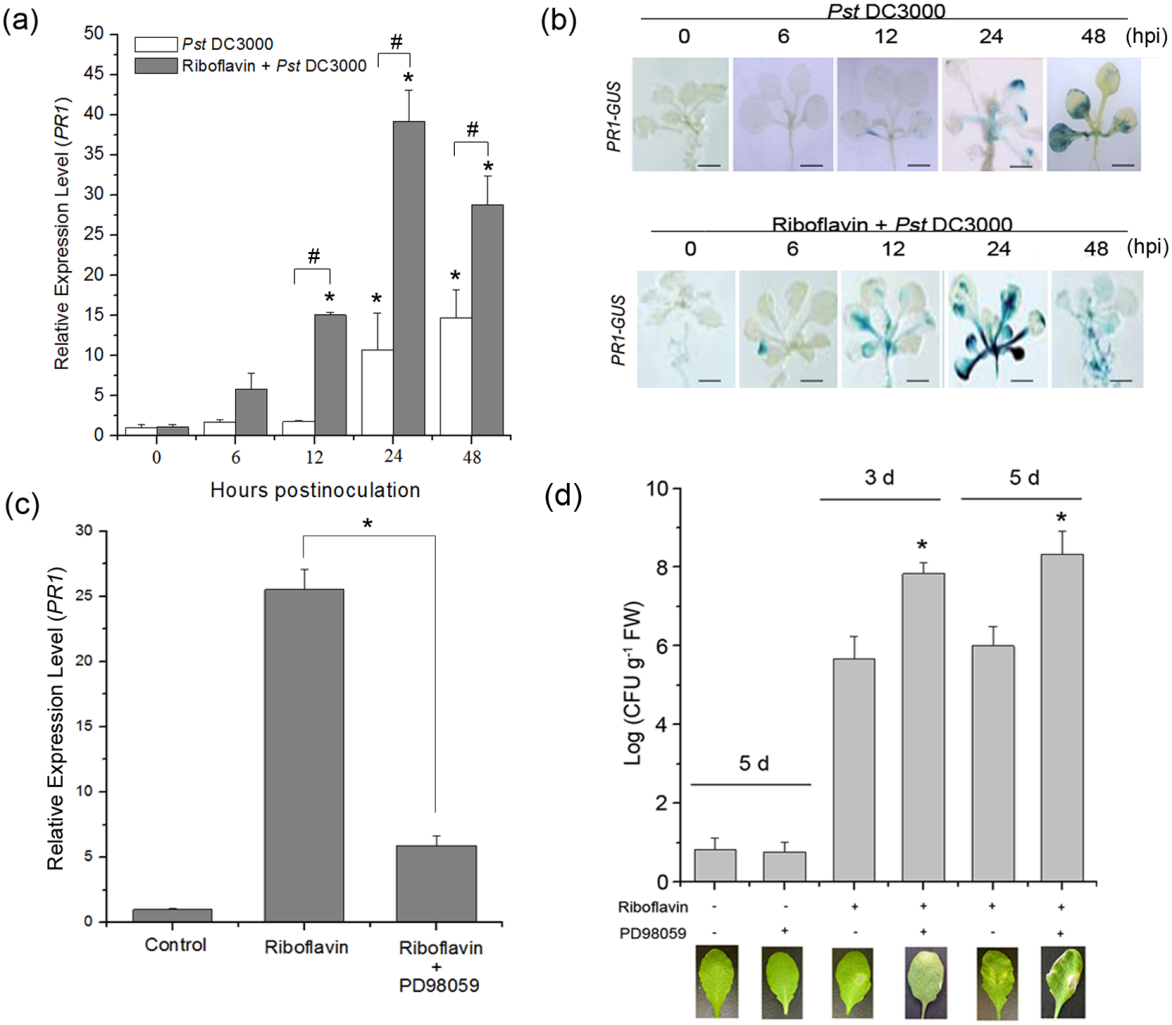
Effect of riboflavin on defense priming in Arabidopsis. (**a**) Real-time quantitative RT-PCR analyses showing induction of *PR1* gene expression in Arabidopsis plants upon infection with *Pst* DC3000 at 0, 6, 12, 24, and 48 hpi. The Arabidopsis ecotype Col-0 plants were sprayed with either water (control) or riboflavin (0.6 mM) in the presence of Silwet L-77 (0.015%) and 4 hours later were challenged with *Pst* DC3000 for the different times. Statistical analysis was performed with Student’s t-test: *, P < 0.05 vs control; #, P < 0.05 vs *Pst* DC3000. (**b**) The transgenic *PR1*:*GUS* seedlings grown on liquid challenged with *Pst* DC3000 for different times, and collected the plants for histochemical GUS staining. (**c**) Effect of MAPK cascade inhibitor (PD98059) on riboflavin-induced *PR1* gene expression in Arabidopsis. Detached leaves were pretreated with or without PD98059 (150 μM), and then treated with 0.6 mM riboflavin for 4 hours and challenged with *Pst* DC3000 for 24 hours. Asterisks indicate significant differences between Riboflavin and Riboflavin + PD98059 (Student’s t-test, P <0.05). (**d**) *Pst* DC3000 growth analysis in PD98059-pretreated detached leaves of wild-type plants after riboflavin. Detached leaves were pretreated with or without PD98059, and then treated with 0.6 mM riboflavin for 4 hours and challenged with Pst DC3000 for 3 days or 5 days. Asterisks indicate significant differences between Riboflavin and Riboflavin + PD98059 (Student’s t-test, P <0.05). Below are representatives of leaves of the indicated genotypes. Data are means ± SD of three replicates.

The transcriptional expression of *PR* gene has been regarded as a molecular indicator for the activation of plant defense pathways [53, 54]. In order to investigate the kinetics of riboflavin action, the expression pattern of *PR1* was analyzed. Inoculation of the plants with *Pst* DC3000 induced the expression of *PR1* at 24 h after inoculation. However, riboflavin treatment induced *PR1* expression from 6 h to 48 h after challenge inoculation and the transcript level of *PR1* was peaked at 24 h, which was more rapid and higher than that observed after pathogen inoculation alone (Fig 2a). Alternatively, the transgenic *PR1*:*GUS* reporter was analyzed for the *PR1* expression. Similar results were detected following the pathogen inoculation (Fig 2b).

To investigate whether MAPK cascades affect the riboflavin-induced priming of defense response in Arabidopsis, PD98059 which is the common inhibitor of MAPK cascade was used. PD98059-pretreatment effectively inhibited the expression of *PR1* at 24 h after challenge inoculation following riboflavin treatment (Fig 2c). In addition, compared to riboflavin-treated plants, pre-treatment with PD98059 caused an increase in bacterial growth 3 d and 5 d after challenge inoculation (Fig 2d). These data indicated that MAPK cascades might be involved in riboflavin-induced resistance and defense gene expression.

### Contribution of MPK3 and MPK6 to riboflavin-induced defense response

**Fig 3:**
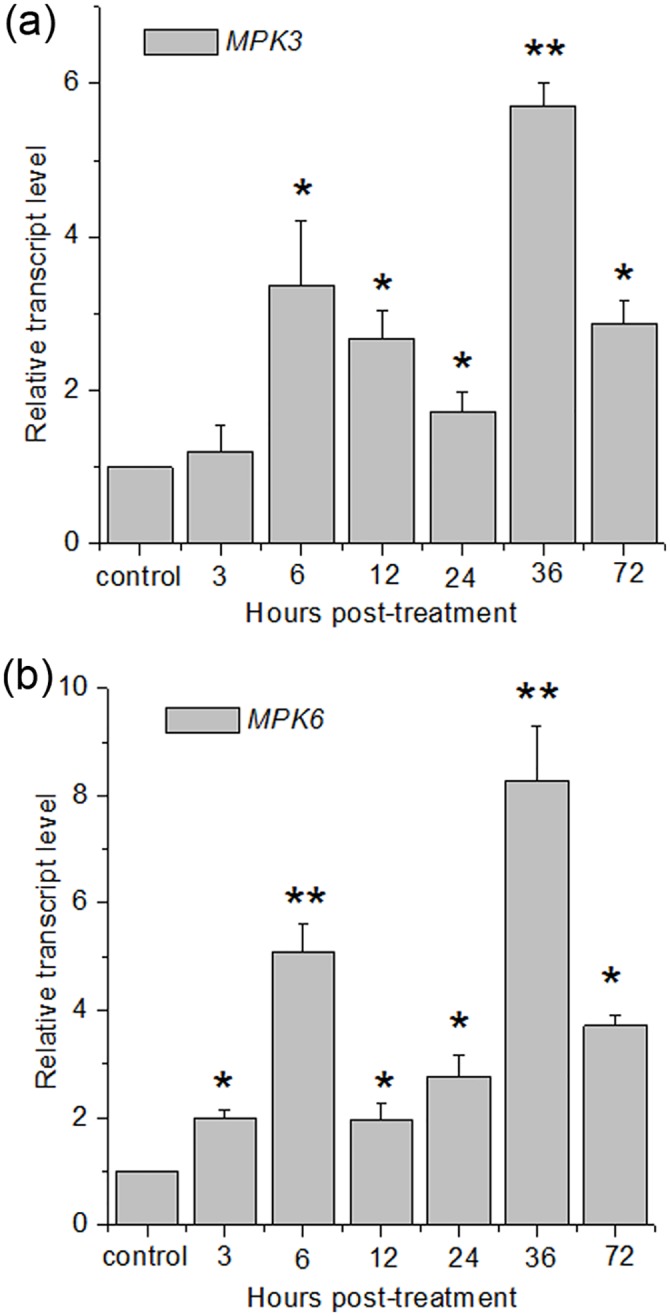
Riboflavin induced expression of *MPK3* and *MPK6* genes. (**a**) The effect of riboflavin on expression level of *MPK3* transcript. The Arabidopsis leaves were harvested at different hours after 0.6 mM riboflavin treatment, and total RNAs were extracted from the detached leaves for the quantitative RT-PCR analysis. Relative transcript levels were normalized with that of *Actin* = 1. (**b**) The effect of riboflavin on expression level of *MPK6* transcript. The experimental method and analyses in **a** were used to evaluate the expression level of MPK6. In (**a**) and (**b**), statistical analysis was performed with Student’s t-test: *, P < 0.05 vs control; **, P < 0.01 vs control. Data are means ± SD of three replicates.

As described above, pretreatment with riboflavin protects Arabidopsis plants against *Pst* DC3000 (Fig 1), and this effect appeared to be associated with MAPK cascades. Then the next investigation was focus on the expression and activation of MPK3 and MPK6. Treatment with riboflavin induced biphasic accumulation of MPK3 and MPK6 transcripts (Fig 3a and 3b). The expression of both genes was transiently up-regulated during the first 6 h, and peaked 36 h after treatment, and riboflavin-induced transcript level of MPK6 was 1.5 times higher than that of MPK3.

**Fig 4:**
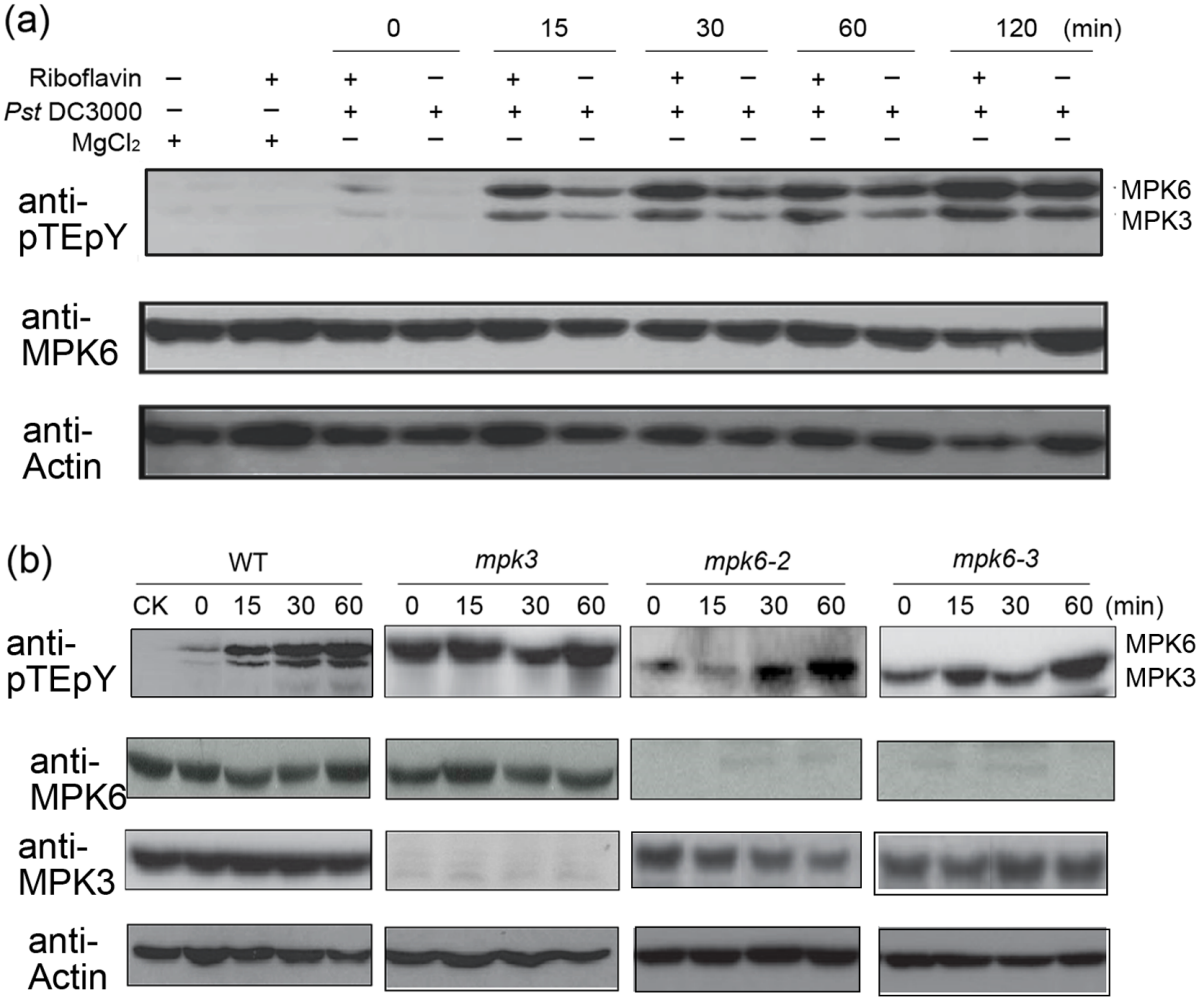
Phosphorylation of MAPKs in riboflavin-primed leaves in response to *Pst* DC3000 challenge. (**a**) Activations of MPK3 and MPK6 in Arabidopsis wild-type (WT) inoculated with *Pst* DC3000. Seedlings of WT were treated with riboflavin or water in the present of Silwet L-77 (0.015%) and then challenged with *Pst* DC3000 or MgCl_2_ and harvested at different time points. Proteins were extracted from treated leaf tissue and 10 mg protein extract was used for SDS-PAGE. Immunoblotting was performed with anti-pTEpY, anti-MPK6 and anti-actin (loading control) antibodies. (**b**) Activations of MPK3 and MPK6 in the *mpk3* and *mpk6* mutant. The amounts of protein loaded are indicated by the actin.

To further investigate the role of MPK3 and MPK6 in riboflavin-induced resistance to Pst DC3000, riboflavin-pretreated plants with induced accumulation of transcripts for MPK3 and MPK6 were challenged with Pst DC3000. Using immunoblot analysis with an anti-phosphoERK 1/2 (anti-pTEpY) antibody, the activation of MPK3 and MPK6 was determined. As shown in Fig 4a and S3 Fig, inoculation with *Pst* DC3000 led to an activation of MPK3 and MPK6 within 120 min in riboflavin-treated plants (+Riboflavin +*Pst* DC3000), and it was more intense than that of in riboflavin-untreated plants (-Riboflavin +*Pst* DC3000). However, the activation of MPK3 and MPK6 were not evident in plants treated with only riboflavin (+Riboflavin +MgCl_2_) and in riboflavin-untreated plants without *Pst* DC3000 inoculation (-Riboflavin +MgCl_2_). No significant increase in kinase activity of MPK3 and MPK6 was observed in Pst DC3000-inoculated leaves of the respective mutants, and immunoblot analysis using anti-MPK3 and anti-MPK6 antibodies confirmed the absence of protein in the respective mutants (Fig 4b; S4 Fig). These results suggested that MPK3 and MPK6 are possible priming components for the riboflavin-induced resistance in Arabidopsis.

### Riboflavin-induced resistance against *Pst* DC3000 is compromised in *mpk3* and *mpk6* mutants

**Fig 5:**
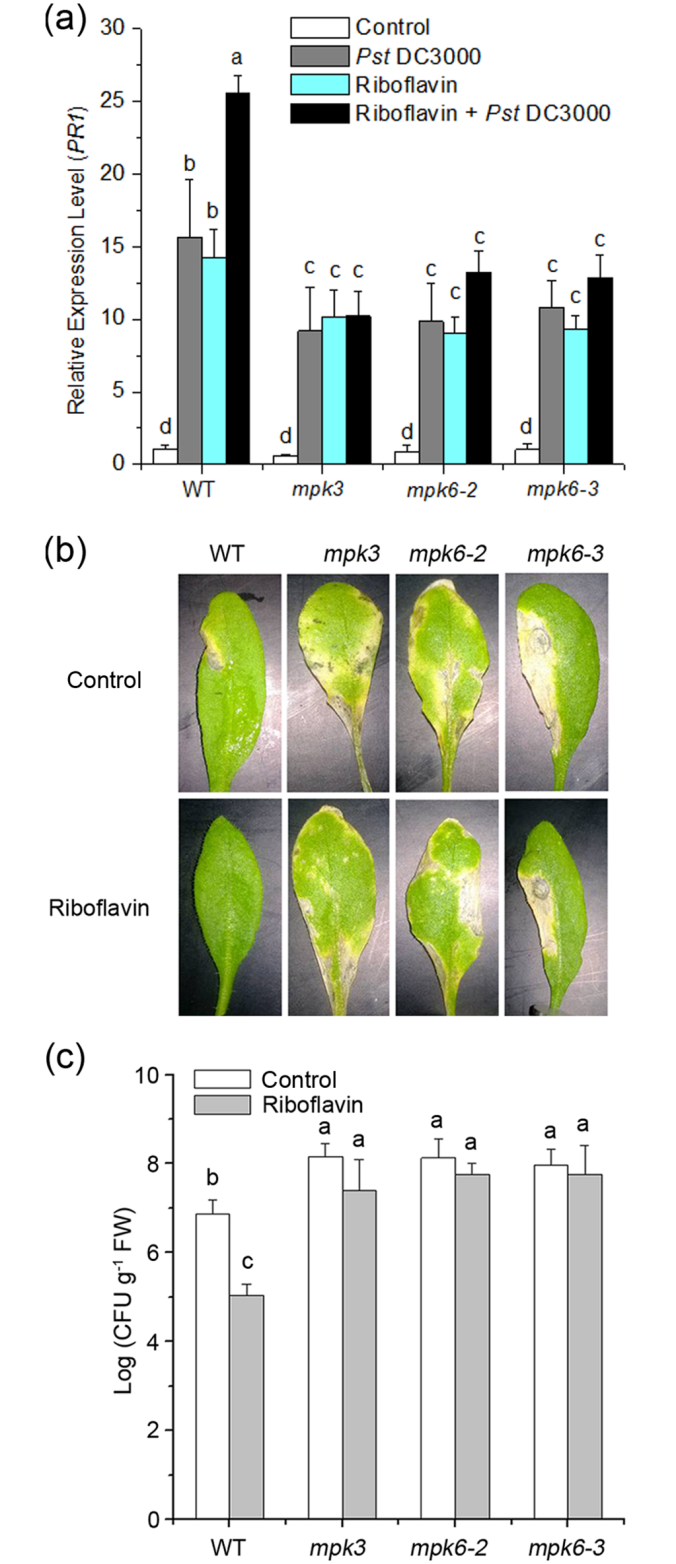
Attenuation of riboflavin-induced resistance in response to *Pst* DC3000 in the *mpk3* and *mpk6* mutant. (**a**) Expression of *PR1* gene is compromised in the *mpk3* and *mpk6* mutant seedlings. 2-week-old WT and single mutant (*mpk3*, *mpk6-2* and *mpk6-3*) seedlings were treated with water or riboflavin, and then inoculated with *Pst* DC3000 for 24 hours. The total RNAs were extracted at indicated times and analyzed for the expression of *PR1* gene. *Actin2* was used as an internal control. Control represents WT and single mutant seedlings were not treated with riboflavin and *Pst* DC3000. Different letters indicate statistically significant differences among the samples (P <0.05; Duncan’s multiple range tests). (**b**) Representative water- or riboflavin-treated leaves at 5 days after inoculation with *Pst* DC3000. The *mpk3* and *mpk6* mutants were significantly more susceptible to *Pst* DC3000 and failed to develop resistance by riboflavin. (**c**) Pathogen growth in WT and single mutant seedlings. Different letters indicate statistically significant differences among the samples (P <0.05; Duncan’s multiple range tests). The experiment was repeated three times with similar results.

To confirm whether both MPK3 and MPK6 play a crucial role in priming and resistance induced by riboflavin, the response to *Pst* DC3000 upon pretreatment with riboflavin was tested in wild-type, *mpk3* and *mpk6* (*mpk6-2* and *mpk6-3*) plants. Leaves from wild-type or mutant plants were pretreated with water or riboflavin and subsequently inoculated with *Pst* DC3000. As shown in Fig 5a, treatment with riboflavin induced expression of *PR1* at 24 h postinoculation in wild-type. In comparison, the expression level of *PR1* was reduced in the *mpk3* and *mpk6* (*mpk6-2* and *mpk6-3*) mutants. As expected, riboflavin-treated wild-type leaves developed significant reduction of bacterial growth with respect to leaves treated with water, whereas the *mpk3* and *mpk6* (*mpk6-2* and *mpk6-3*) mutants were significantly more susceptible to *Pst* DC3000 and also failed to develop resistance by riboflavin (Fig 5b and 5c). In sum, these results suggested that MPK3 and MPK6 are required for riboflavin-induced resistance for defense response in Arabidopsis infected with *Pst* DC3000.

### Riboflavin-induced resistance mediating MPK3 and MPK6 through the ROS- and Ca^2+^-dependent signaling pathways

**Fig 6:**
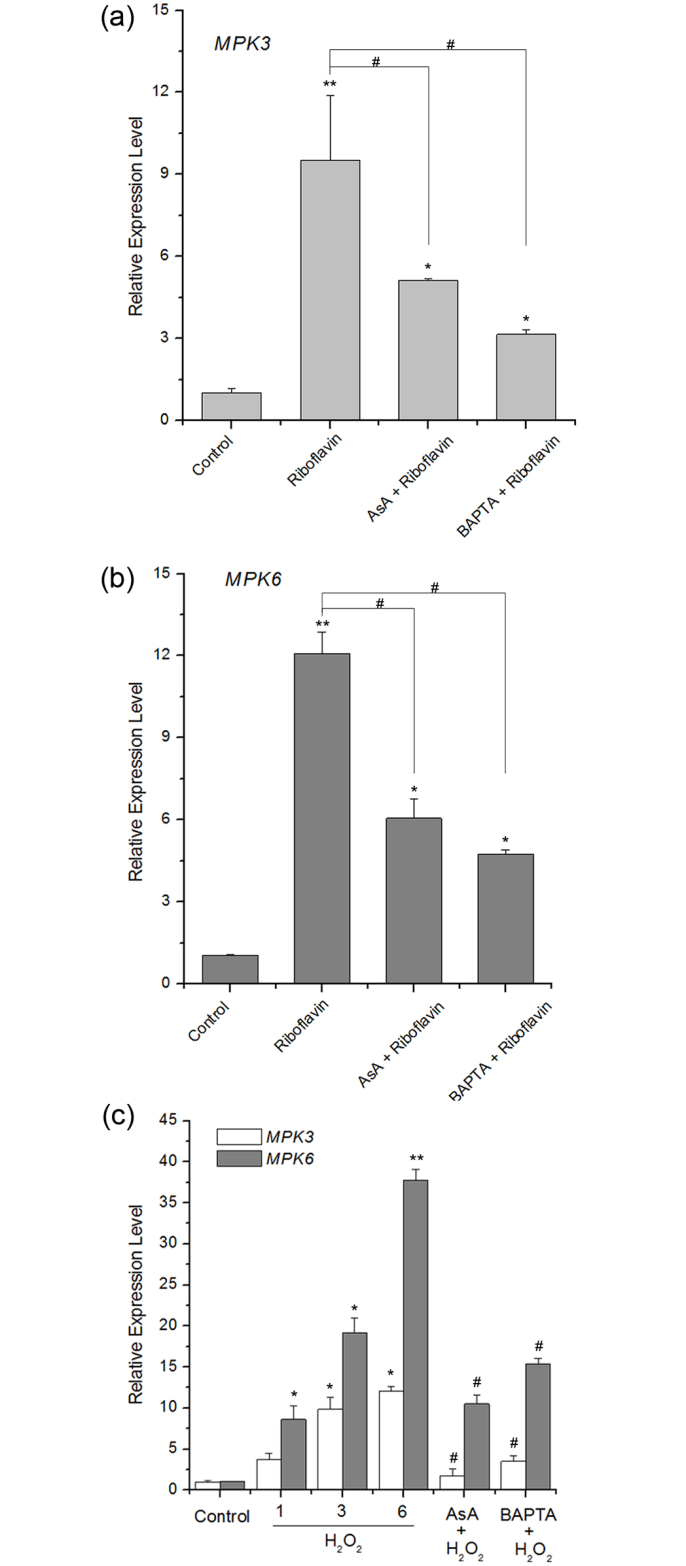
Roles of H_2_O_2_ and Ca^2+^ in riboflavin-induced expression of *MPK3* and *MPK6* genes in Arabidopsis seedlings. (**a**) and (**b**) Real-time quantitative RT-PCR analyses showing the expression of *MPK3* and *MPK6* genes measured in WT plants with AsA (1.5 mM) or BAPTA (1 mM) pretreatment in response to *Pst* DC3000 challenge in 0.6 mM riboflavin-treated Arabidopsis seedlings or not. *Actin2* was used as an internal control. Asterisks indicate significant differences to control (student’s t-test: *p < 0.05, **p < 0.01), and #, P < 0.05 vs Riboflavin. (**c**) Time course analysis of *MPK3* and *MPK6* genes expression in response to exogenous H_2_O_2_ treatment. Seedlings were pretreated with AsA (1.5 mM) or BAPTA (1 mM), and then incubated with H_2_O_2_. Samples were collected at the indicated time points. Asterisks indicate significant differences to control (student’s t-test: *p < 0.05, **p < 0.01), and #, P < 0.05 vs H_2_O_2_-treated samples at 6 h.

**Fig 7:**
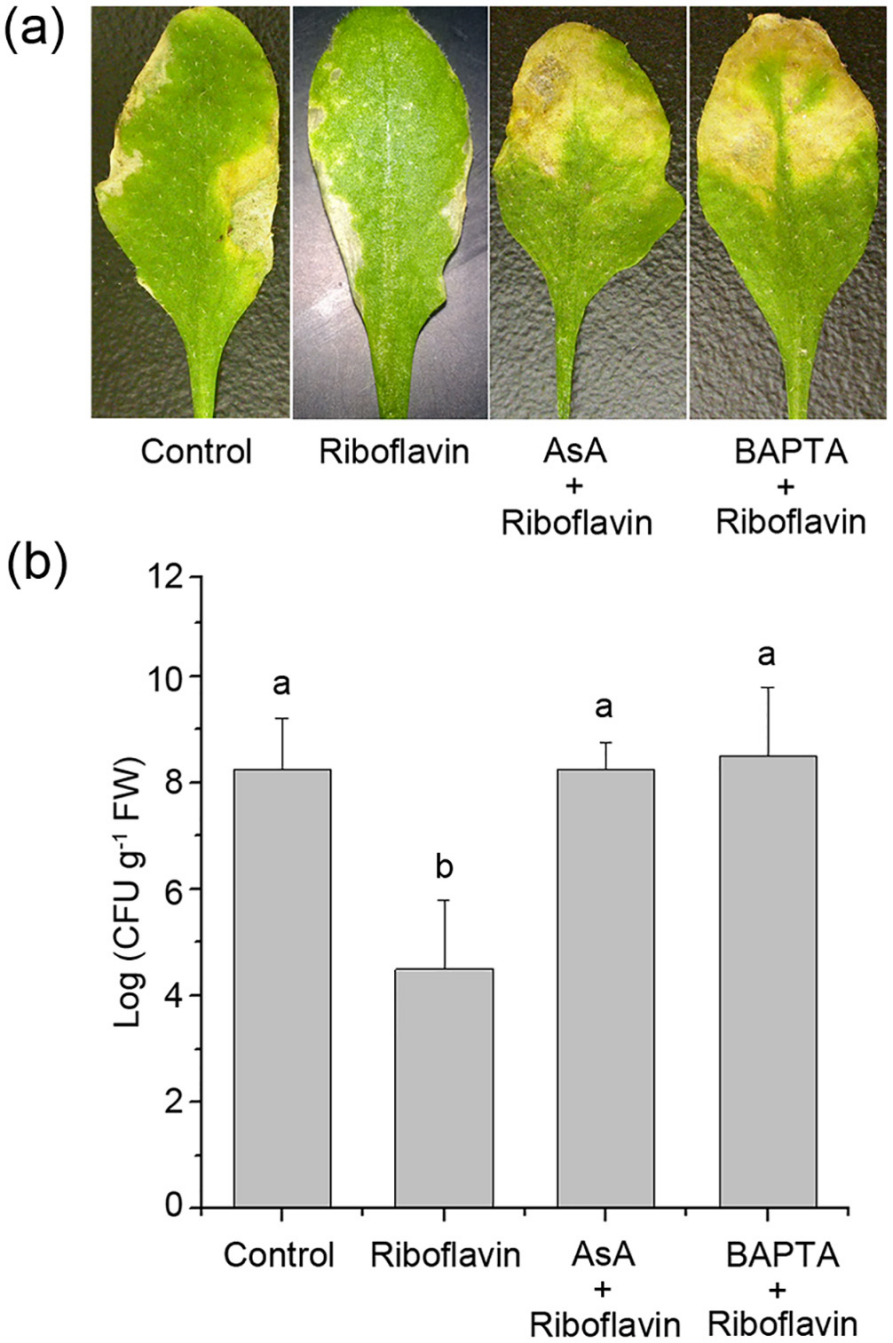
Effects of AsA and BAPTA on disease development in Arabidopsis treated with riboflavin and challenged with *Pst* DC3000. (**a**) The necrotic lesions on representative Arabidopsis leaves at 5 days after infected by *Pst* DC3000 in AsA- or BAPTA-pretreated plants. Detached leaves were pretreated with AsA (1.5 mM) or BAPTA (1 mM), and then treated with 0.6 mM riboflavin for 4 hours and challenged with *Pst* DC3000 for 5 days. (**b**) *Pst* DC3000 growth analysis in AsA- or BAPTA-pretreated detached leaves of wild-type after riboflavin. FW, fresh weight. Letters indicate statistically significant differences among the samples (P <0.05; Duncan’s multiple range tests). Data are means ± SD of three replicates.

Previous studies suggested that ROS and Ca^2+^, as important signal messengers in plants cells, might function in the upstream activation of MAPK cascade under stimuli [12, 39, 51]. Furthermore, among the earliest cellular events in plant-pathogens interactions, H_2_O_2_ and ion fluxes across the membrane, such as Ca^2+^, play important roles [39, 55]. To determine whether ROS and Ca^2+^ signaling pathways are involved in riboflavin-induced resistance, we tested the effect of riboflavin on the transcript levels of MPK3 and MPK6. As shown in Fig 6a and 6b, upon infection by *Pst* DC3000, the transcript levels of MPK3 and MPK6 were both up-regulated. However, pretreatment with ROS scavenger AsA and the intracellular Ca^2+^ scavenger BAPTA-AM effectively arrested riboflavin-induced *MPK3* and *MPK6* expression, respectively. Furthermore, both *MPK3* and *MPK6* genes were induced in a time dependent manner in response to 1.5 mM H_2_O_2_, and H_2_O_2_-induced expression of these two genes was inhibited by AsA. Most importantly, treatment with BAPTA-AM before challenge with H_2_O_2_ significantly reduced *MPK3* and *MPK6* genes transcripts (Fig 6c). In addition, resistance by riboflavin inhibited disease progression, including the necrotic lesion and bacterial growth, in response to bacterial pathogen *Pst* DC3000 in Arabidopsis. However, pretreatment with AsA or BAPTA-AM interdicted these effects (Fig 7a and 7b).

## Discussion

In this study, the effectiveness and potential molecular mechanisms of riboflavin-induced resistance against pathogen *Pst* DC3000 have been investigated. The results shown in this work might provide evidence for the cellular signaling cascade which MPK3 and MPK6 were responsible for riboflavin-induced defense response.

Riboflavin endows Arabidopsis with resistance to pathogen *Pst* DC3000 and induces priming for pathogen defense without a direct effect on the causal pathogen [19, 20]. Priming, as one of the various forms of induced resistance in plants and animals, enables cells to show a faster and stronger activation of defense upon a stress stimulus [3, 56–58]. The results presented here showed that riboflavin induced resistance in Arabidopsis to infection by *Pst* DC3000. In addition, riboflavin did not result in any remarkable alterations and phytotoxicity to *Pst* DC3000 at any of the tested concentrations in the Arabidopsis plants (Fig 1). As reported by others, riboflavin affects defense-related gene expression (PR) in tobacco and Arabidopsis [19, 20]. Some chemicals, such as non-protein amino acid BABA [11] and a B-complex vitamin thiamine [13], which both activate resistance, also induce the transient expression of defense-related genes, although their acting sites are different. In our work, it was found that inoculation of *Pst* DC3000 without riboflavin pretreatment induced transient *PR1* gene expression within 24 to 48 hours. However, following infection by the pathogen *Pst* DC3000, *PR1* gene was rapidly and strongly expressed in riboflavin-pretreated plants (Fig 2). Just as a phenomenon in mammalian monocytes [59], riboflavin triggers the priming defense in Arabidopsis plants and alters the plant into a highly competent state in the absence of detectable variations [3]. Thus, this priming of riboflavin-induced defense-related gene may allow the plant to react more effectively to a subsequent invader, such as *Pst* DC3000.

As priming for enhanced *PR1* expression in riboflavin-induced defense, riboflavin is capable of triggering a resistance signaling process [19, 20]. However, the upstream signaling cascades of riboflavin-induced defense-related gene expression remain unclear. MAPKs cascades can be activated by extra- and intracellular stimuli and play essential roles in the process whereby extracellular stimuli are transmited into intracellular responses while at the same time amplifed the signal [21, 23–25, 28, 60]. What is more, MAPKs cascades also function as negative regulators in stress responses. For example, a mutation in MKK9 exhibits enhanced stress tolerance [61, 62]. Among these MAPK proteins, MPK3 and MPK6 are activated by various stimulli, including pathogen, UV-B stress, and plant hormones or their functional analogs [32, 63, 64]. In this work, we demonstrated that MAPK cascades participated in the riboflavin-induced resistance against *Pst* DC3000 challenge (Fig 2c and 2d). In riboflavin-pretreated Arabidopsis, the activations of MPK3 and MPK6 were proved to be responsible for the up-regulation of the defense-related gene (PR1) and the subsequent enhanced resistance (Figs 4 and 5). In the *mpk3* and *mpk6* mutants (*mpk3*, *mpk6-2* and *mpk6-3*), which lack detectable transcripts of *MPK3* and *MPK6*, respectively (S1 Fig.), riboflavin-induced priming defense was markedly reduced at the indicated time (Fig 5a and 5b). However, it is not clear whether these activated MPKs have redundant or separate functions in response to riboflavin. Unfortunately, simultaneous knockout of *MPK3* and *MPK6* is embryo-lethal [65], such *mpk3mpk6* double mutation is not available to more strictly address the roles of MPK3 and MPK6 in stress-induced priming. In addition, although MPK3 and MPK6 seem to have redundant functions, distinct roles are suggested in recent evidences [33, 37].

ROS and Ca^2+^, as the key signaling molecules in plant cells, function upstream of activation of MAPKs [39, 66]. Zhang et al. [19] reported that potentiated ROS production is required for resistance by riboflavin in response to *Pst* DC3000 challenge. In contrast, riboflavin alone or *Pst* DC3000 inoculation does not induce ROS production at the same time, which is similar to the phenomena in thiamine–induced priming defense [13]. What is more, among the earliest cellular events in plant-pathogens interactions, H_2_O_2_ and ion fluxes across the membrane, such as Ca^2+^, also play important roles [39, 55]. Accordingly, ROS accumulation and Ca^2+^ should be one of the defense mechanisms of priming. Our work presented a cellular signal cascade, composed of endogenous ROS production, [Ca^2+^]_cyt_ rise, and MPK3 and MPK6 induction in response to pathogen (S6, S7 and S8 Figs; Fig 6). After challenge inoculation of the riboflavin-pretreated plants with *Pst* DC3000, the riboflavin-induced accumulation of MPK3 and MPK6 transcripts and proteins was prevented by AsA and BAPTA, an H_2_O_2_ scavenger and [Ca^2+^]_cyt_ scavenger, respectively (Fig 6; S5 Fig), this was accompanied by abolition of disease progression, including the necrotic lesion and bacterial growth (Fig 7). Furthermore, exogenous H_2_O_2_-induced expression of *MPK3* and *MPK6* transcripts was markedly reduced in the present of BAPTA (Fig 6). Hence, results here suggest that ROS and Ca^2+^, which functioned in the upstream activation of MPK3 and MPK6, are required for resistance by riboflavin.

Recently, NPR1, as a regulator protein, is required in development of induced resistance induced by pathogen infection [67]. Dong and Beer [20] and Zhang *et al*. [19] reported that riboflavin functions as a plant defense activator, and induces disease resistance which requires a functional NPR1 in response to virulent *Pst* DC3000, which is just like the effect of thiamine [12,13]. In this work, the *npr1* mutant showed more developed chlorotic lesions compared with WT, and riboflavin-pretreated *npr1* mutant exhibited no significant improvement on disease progression, including bacterial growth (S9 Fig). NPR1 moves into the nucleus, where it activates the expression of *PR1* gene [68] via TGA transcription factors. Riboflavin treatment induced *PR1* expression after challenge inoculation in WT plants. However, riboflavin pre-treatment did not promote increased *PR1* transcript in *npr1* mutant (S10 Fig). Further, the link of MAPKs and NPR1 after *Pst* DC3000 inoculation was investigated. Inoculation with *Pst* DC3000 promoted expression of NPR1 protein in WT plants, whereas the expression of NPR1 was nearly arrested in the *mpk3* and *mpk6* mutants (S11 Fig). These data indicate that riboflavin induces defense priming through an NPR1-dependent signaling pathway in response to *Pst* DC3000 and riboflavin-induced this MAPKs signal module may operate upstream of the NPR1 regulator. However, the concrete interaction between MAPKs and NPR1 is still needed to be study in future work.

**Fig 8:**
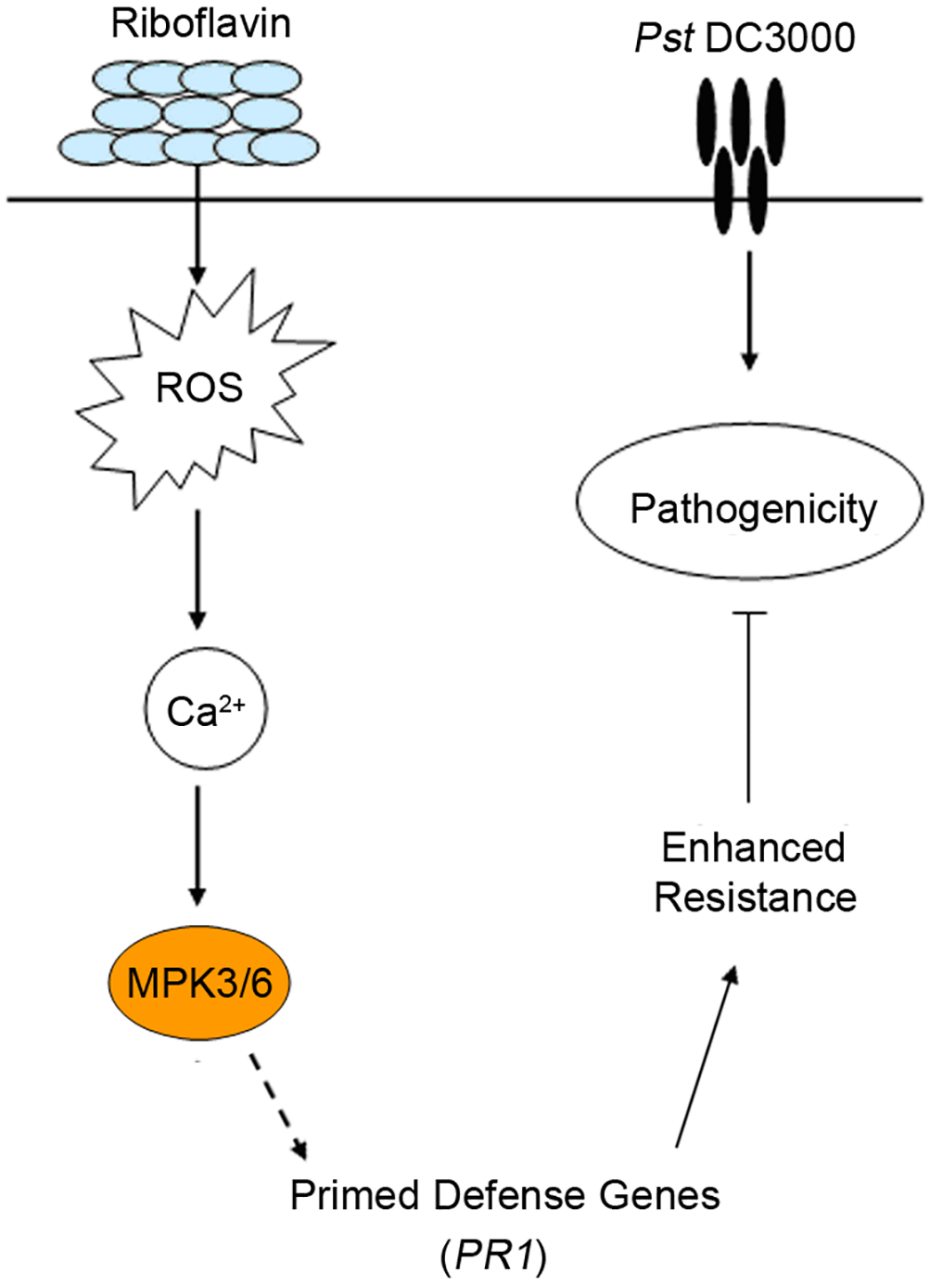
Proposed model for the contribution of MPK3 and MPK6 to riboflavin-induced disease resistance to bacterial pathogen *Pst* DC3000. Riboflavin triggers activations of MPK3 and MPK6 in the defense response via ROS- and Ca^2+^-dependent signaling pathways. The induction by riboflavin is associated with priming for enhanced expression of defense-related gene (*PR1*) that becomes apparent upon infection by pathogen *Pst* DC3000, which allows the Arabidopsis plants to react more effectively to *Pst* DC3000.

Taken together, our results demonstrated the contribution of MPK3 and MPK6 to riboflavin-induced resistance to pathogen *Pst* DC3000 and riboflavin exerted its effect via ROS- and Ca^2+^-dependent signaling pathways. The results further demonstrated that priming defense and its associated molecular defense mechanisms were induced by riboflavin. According to our experimental results, a potential cascade of cellular events occurred during riboflavin-induced priming defense (Fig 8). Riboflavin increased levels of *MPK3* and *MPK6* transcript and transient expression of *PR1* gene. Upon infection by *Pst* DC3000, the molecular defense-related responses, including the expression of *PR1* transcript and the activities of MPK3 and MPK6, were strongly enhanced in riboflavin-applied Arabidopsis. These findings add to our understanding of the signaling pathways in disease resistance mediated by riboflavin. In conclusion, defense responses induced by riboflavin could be one of the most economical and effective resistances, just like the resistance induced by thiamine and BABA [9, 12], providing a novel disease control strategy and satisfied environmental regulations.

## Supporting Information

S1 Fig Characterization of *mpk3* and *mpk6* mutants using semi-quantitative RT-PCR. (DOCX)

S2 Fig Biosynthesis pathway of riboflavin, FMN, and FAD in plants. (DOCX)

S3 Fig Quantitative analysis of activation (p-MPK6 and p-MPK3) and protein (MPK6) of MPK3/6 proportion shown in Fig 4a. (DOCX)

S4 Fig Quantitative analysis of activation (p-MPK6 and p-MPK3) and protein (MPK6 and MPK3) of MPK3/6 proportion shown in Fig 4b. (DOCX)

S5 Fig Roles of ROS/Ca^2+^ in riboflavin-induced activation of MAPKs. (DOCX)

S6 Fig Effect of priming by riboflavin on O2- and H_2_O_2_ generation in Arabidopsis upon *Pst* DC3000 inoculation. (DOCX)

S7 Fig Effect of AsA on H_2_O_2_ level in riboflavin-pretreated Arabidopsis upon *Pst* DC3000 inoculation. (DOCX)

S8 Fig Effect of defence priming by riboflavin on Ca^2+^ level in Arabidopsis upon *Pst* DC3000 inoculation. (DOCX)

S9 Fig Effect of riboflavin on disease progression in *npr1* mutant. (DOCX)

S10 Fig Effect of riboflavin on *PR1* gene expression in *npr1* mutant. (DOCX)

S11 Fig Effect of riboflavin on the expression of NPR1 protein in WT, *mpk3*, and *mpk6* mutant in response to *Pst* DC3000. (DOCX)

S1 Table Primers for several genes. (DOCX)
