# Supplementary material for: Riboflavin-Induced Disease Resistance Requires the Mitogen-Activated Protein Kinases 3 and 6 in Arabidopsis thaliana
Source: PLoS One. 2016 Apr 7;11(4):e0153175. doi: 10.1371/journal.pone.0153175 (PMC4824526; doi:10.1371/journal.pone.0153175)
Supplement: S1 Fig — (DOCX) [file pone.0153175.s001.docx]

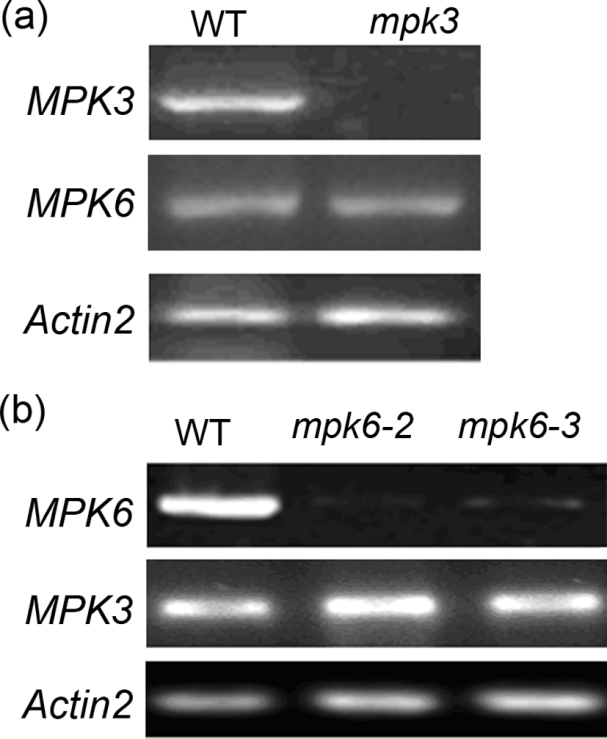


**S1 Fig.**

**S1 Fig. Characterization of *mpk3* and *mpk6* mutants using semi-quantitative RT-PCR.** The total RNAs were extracted from the leaves of 3-week-old Arabidopsis wild-type and mutant (*mpk3*, *mpk6-2* and *mpk6-3*) seedlings and analyze the transcripts by the **semi-quantitative** RT-PCR, and the *Actin2* was analyzed as standard. *MPK3* and *MPK6* expression in WT and *mpk3* mutant (**a**) or *mpk6-2* and *mpk6-3* mutants (**b**).
