# Supplementary figures and images for: Riboflavin-Induced Disease Resistance Requires the Mitogen-Activated Protein Kinases 3 and 6 in Arabidopsis thaliana
Source: PLoS One. 2016 Apr 7;11(4):e0153175. doi: 10.1371/journal.pone.0153175 (PMC4824526; doi:10.1371/journal.pone.0153175)

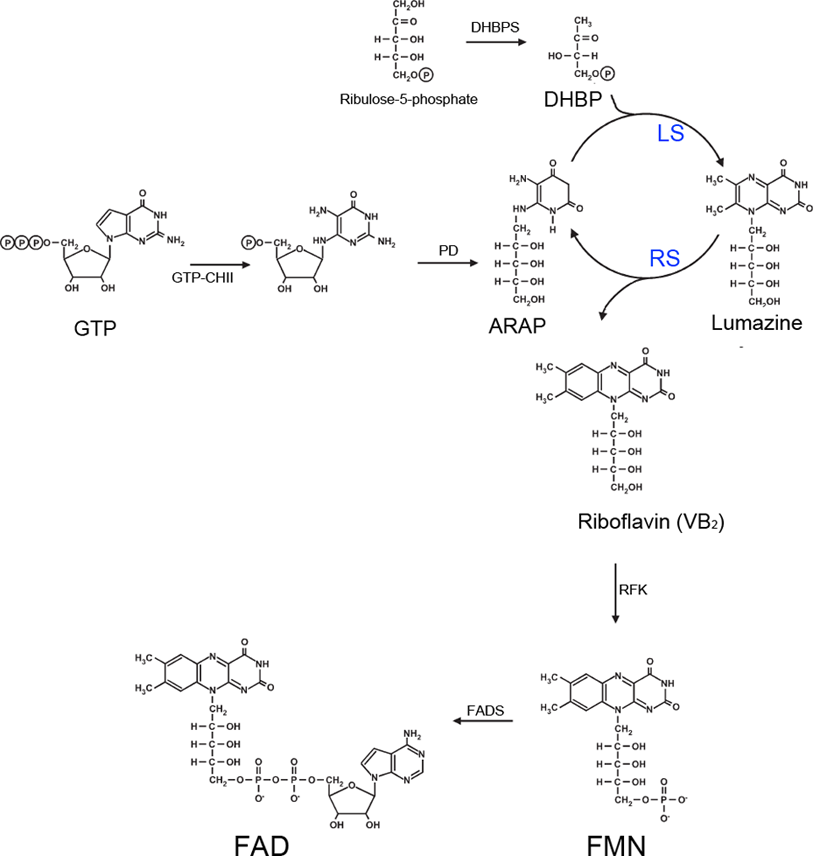


**S2 Fig.**

**S2 Fig. Biosynthesis pathway of riboflavin, FMN, and FAD in plants.**

Supplement: S2 Fig — (DOCX) [file pone.0153175.s002.docx]
