# Supplementary material for: Riboflavin-Induced Disease Resistance Requires the Mitogen-Activated Protein Kinases 3 and 6 in Arabidopsis thaliana
Source: PLoS One. 2016 Apr 7;11(4):e0153175. doi: 10.1371/journal.pone.0153175 (PMC4824526; doi:10.1371/journal.pone.0153175)
Supplement: S3 Fig — (DOCX) [file pone.0153175.s003.docx]

**
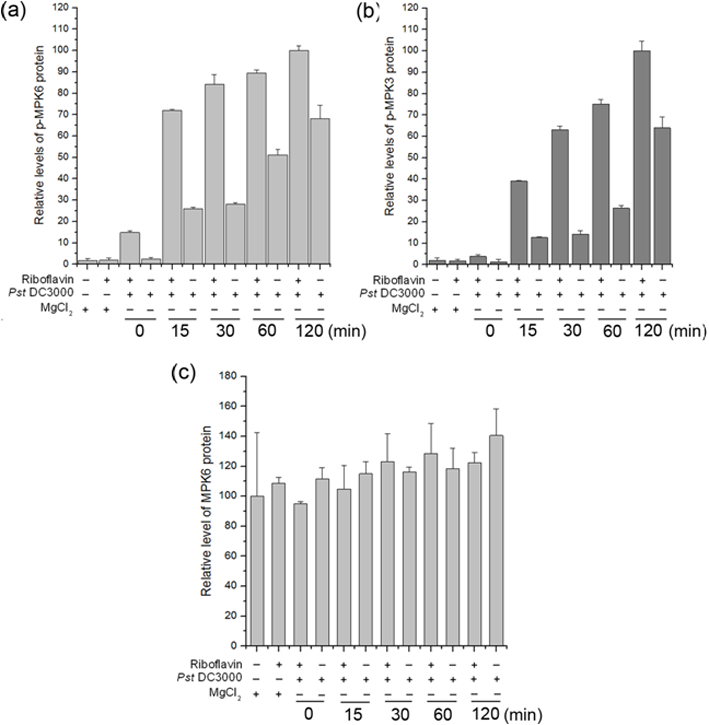
**

**S3 Fig.**

**S3 Fig. Quantitative analysis of activation (p-MPK6 and p-MPK3) and protein (MPK6) of MPK3/6 proportion shown in Figure 4a.** (**a**) and (**b**), Quantitative analysis of MPK6 phosphorylation and MPK3 phosphorylation, respectively. (**c)** Quantitative analysis of the levels of MPK6 protein. Data are means ± SD of three replicates**.**
