# Supplementary material for: Riboflavin-Induced Disease Resistance Requires the Mitogen-Activated Protein Kinases 3 and 6 in Arabidopsis thaliana
Source: PLoS One. 2016 Apr 7;11(4):e0153175. doi: 10.1371/journal.pone.0153175 (PMC4824526; doi:10.1371/journal.pone.0153175)
Supplement: S4 Fig — (DOCX) [file pone.0153175.s004.docx]

**
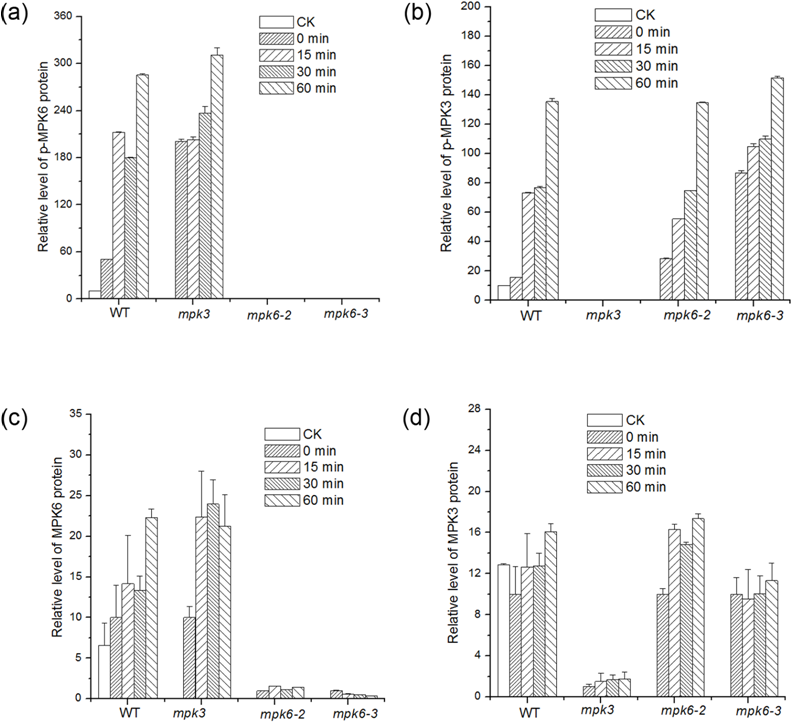
**

**S4 Fig.**

**S4 Fig. Quantitative analysis of activation (p-MPK6 and p-MPK3) and protein (MPK6 and MPK3) of MPK3/6 proportion shown in Figure 4b.**  (**a**) and (**b**), Quantitative analysis of MPK6 phosphorylation and MPK3 phosphorylation, respectively. (**c**) and (**d**) Quantitative analysis of the levels of MPK6 and MPK3 proteins. Data are means ± SD of three replicates**.**
