# Supplementary material for: Riboflavin-Induced Disease Resistance Requires the Mitogen-Activated Protein Kinases 3 and 6 in Arabidopsis thaliana
Source: PLoS One. 2016 Apr 7;11(4):e0153175. doi: 10.1371/journal.pone.0153175 (PMC4824526; doi:10.1371/journal.pone.0153175)
Supplement: S5 Fig — (DOCX) [file pone.0153175.s005.docx]

**
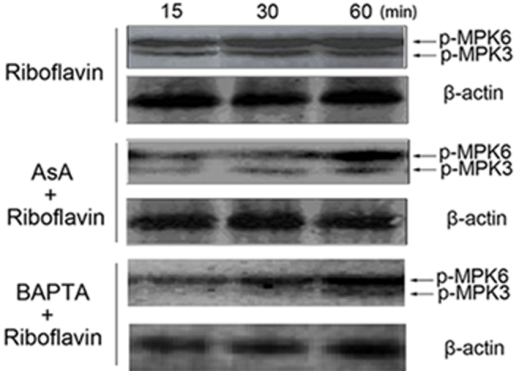
**

**S5 Fig.**

**S5 Fig. Roles of ROS/Ca^2+^ in riboflavin-induced activation of MAPKs. The** MPK3/6 activations were measured in WT plants with AsA (1.5 mM) or BAPTA (1 mM) pretreatment in response to *Pst* DC3000 challenge in 0.6 mM riboflavin-treated Arabidopsis seedlings or not. The protein was extracted at the times indicated and 10 mg protein extract was used for SDS-PAGE, and analysis by Western blot. The amounts of protein loaded are indicated by β-actin.
