# Supplementary material for: Riboflavin-Induced Disease Resistance Requires the Mitogen-Activated Protein Kinases 3 and 6 in Arabidopsis thaliana
Source: PLoS One. 2016 Apr 7;11(4):e0153175. doi: 10.1371/journal.pone.0153175 (PMC4824526; doi:10.1371/journal.pone.0153175)
Supplement: S6 Fig — (DOCX) [file pone.0153175.s006.docx]

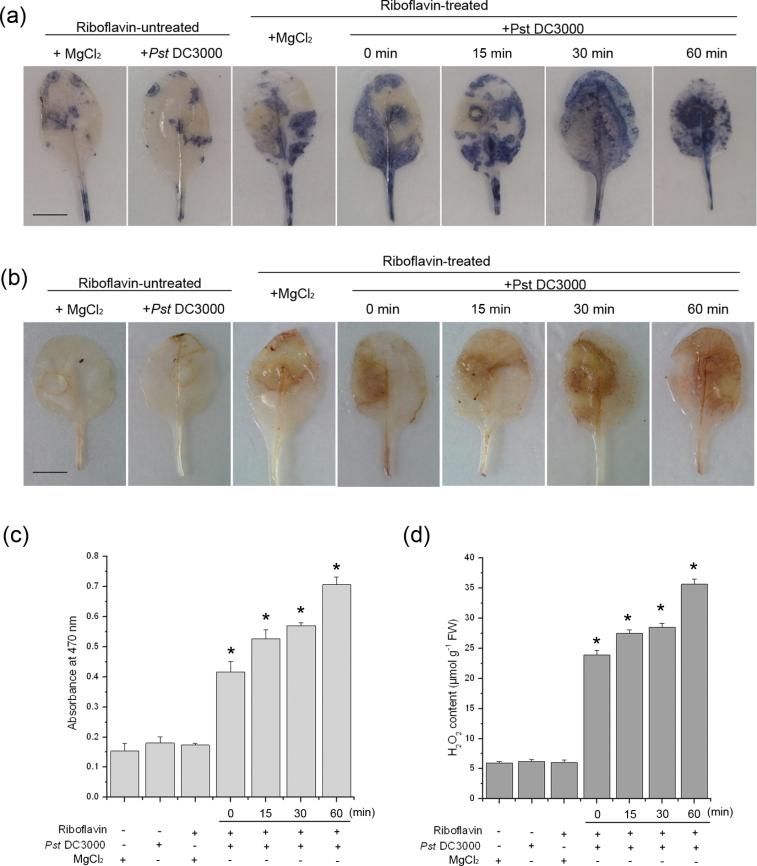


**S6 Fig.**

**S6 Fig. Effect of priming by riboflavin on O_2_^-^ and H_2_O_2_ generation in Arabidopsis upon *Pst* DC3000 inoculation.** (**a**) *In sute* detection of leaf O_2_^-^ accumulation. Seedlings of Arabidopsis ecotype Col-0 were sprayed with either water or riboflavin (0.6 mM) in the presence of Silwet L-77 (0.015%) and then challenged with MgCl_2_ or *Pst* DC3000, and harvested at different time points. Bar, 0.5 cm. (**b**) *In situ* detection of leaf H_2_O_2_. DAB stain was used to detect the H_2_O_2_ in leaves. Bar, 0.5 cm. (**c**) Effects of riboflavin and/or *Pst* DC3000 inoculation on O_2_^-^ accumulation. (**d**) Effects of riboflavin and/or *Pst* DC3000 inoculation on H_2_O_2_ accumulation. Asterisks indicate significant differences between Riboflavin + *Pst* DC3000 treatment and Riboflavin or *Pst* DC3000 treatment alone (Student’s t-test, P <0.05). Data are means ± SD of five replicates.
