# Supplementary material for: Riboflavin-Induced Disease Resistance Requires the Mitogen-Activated Protein Kinases 3 and 6 in Arabidopsis thaliana
Source: PLoS One. 2016 Apr 7;11(4):e0153175. doi: 10.1371/journal.pone.0153175 (PMC4824526; doi:10.1371/journal.pone.0153175)
Supplement: S7 Fig — (DOCX) [file pone.0153175.s007.docx]

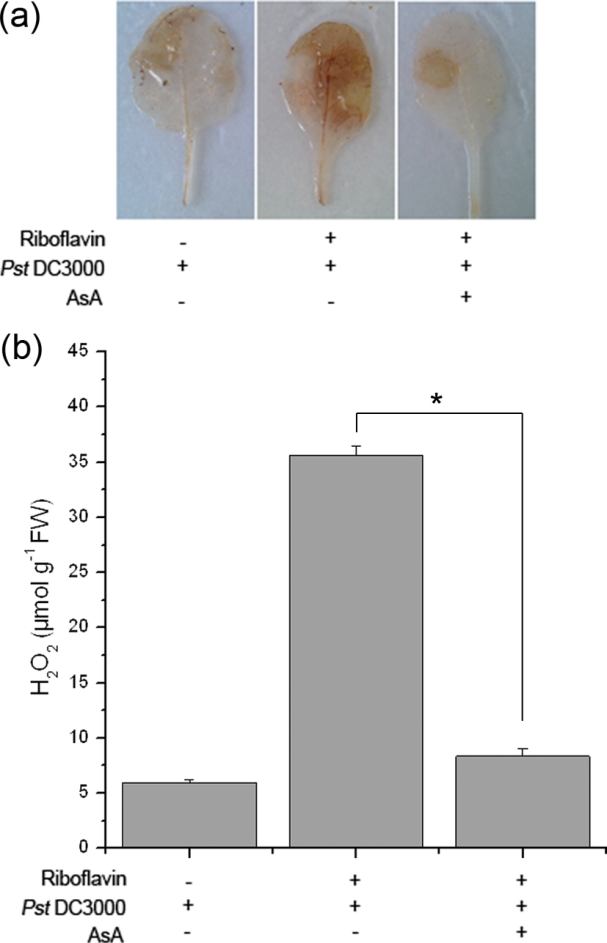


**S7 Fig.**

**S7 Fig. Effect of AsA on H_2_O_2_ level in riboflavin-pretreated Arabidopsis upon *Pst* DC3000 inoculation.**  **(a)** *In situ* detection of leaf H_2_O_2_ in response to AsA (1.5 mM) in Arabidopsis treated with riboflavin and challenged with *Pst* DC3000. (**b**) Effects of exogenous application of AsA on H_2_O_2_ accumulation in Arabidopsis treated with riboflavin and challenged with *Pst* DC3000. Asterisk indicates significant difference between Riboflavin + *Pst* DC3000 and AsA + Riboflavin + *Pst* DC3000 (Student’s t-test, P <0.05). Data are means ± SD of five replicates.
