# Supplementary material for: Riboflavin-Induced Disease Resistance Requires the Mitogen-Activated Protein Kinases 3 and 6 in Arabidopsis thaliana
Source: PLoS One. 2016 Apr 7;11(4):e0153175. doi: 10.1371/journal.pone.0153175 (PMC4824526; doi:10.1371/journal.pone.0153175)
Supplement: S8 Fig — (DOCX) [file pone.0153175.s008.docx]

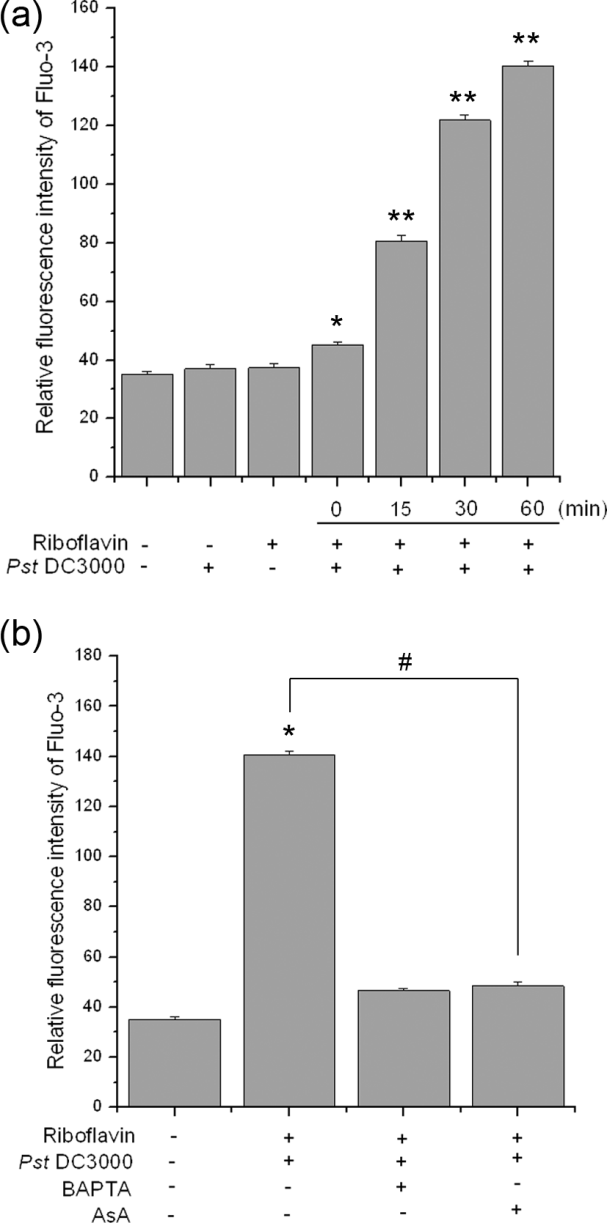


**S8 Fig.**

**S8 Fig. Effect of defence priming by riboflavin on Ca^2+^ level in Arabidopsis upon *Pst* DC3000 inoculation.** (**a**) Riboflavin-induced elevation of Ca^2+^ level in *Pst* DC3000-infected wild-type leaves. The leaves of Arabidopsis ecotype Col-0 plants were sprayed with either water or riboflavin (0.6 mM) in the presence of Silwet L-77 (0.015%) and then challenged with MgCl_2_ or *Pst* DC3000, and harvested at different time points. The changes of Ca^2+^ level was detected using fluorescence probe Fluo-3, and the fluorescence intensity of Fluo-3 was analyzed with fluorescence spectronmeter. Asterisks indicate significant differences to riboflavin or *Pst* DC3000 treatment alone (student’s t-test: *p < 0.05, **p < 0.01). (**b**) The role of BAPTA and AsA on the riboflavin-induced Ca^2+^ level in *Pst* DC3000-inoculated wild-type leaves. The changes of Ca^2+^ level were measured in wild-type plants with AsA (1.5 mM) or BAPTA (1 mM) pretreatment in response to *Pst* DC3000 challenge in 0.6 mM riboflavin-treated Arabidopsis seedlings or not. Asterisk indicates significant difference to BAPTA pre-treatment (student’s t-test: *p < 0.05), and #, P < 0.05 vs AsA pre-treatment.
