# Supplementary material for: Riboflavin-Induced Disease Resistance Requires the Mitogen-Activated Protein Kinases 3 and 6 in Arabidopsis thaliana
Source: PLoS One. 2016 Apr 7;11(4):e0153175. doi: 10.1371/journal.pone.0153175 (PMC4824526; doi:10.1371/journal.pone.0153175)
Supplement: S9 Fig — (DOCX) [file pone.0153175.s009.docx]

**
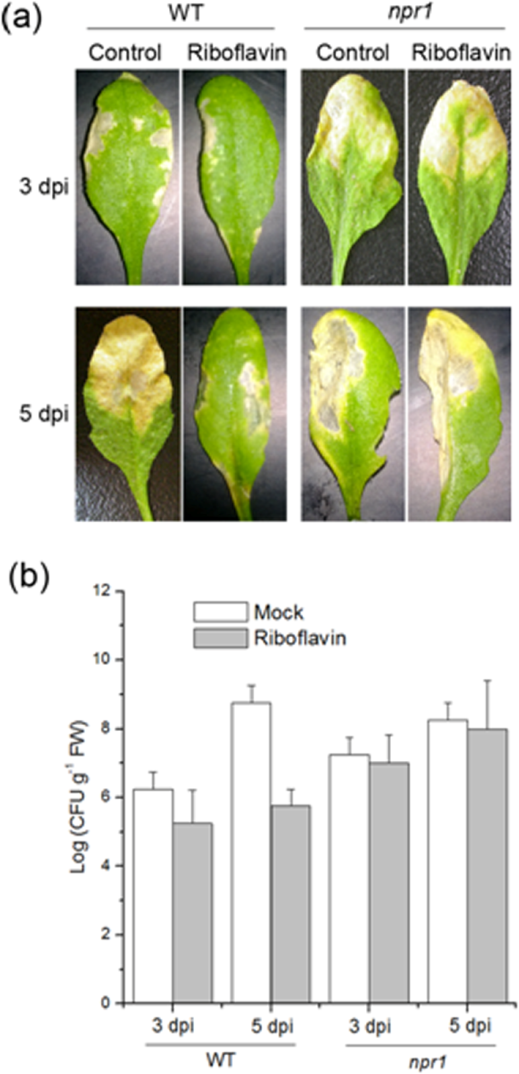
**

**S9 Fig.**

**S9 Fig. Effect of riboflavin on disease progression in *npr1* mutant.**  (**a**) Arabidopsis ecotype Col-0 plants and *npr1* mutant were sprayed with either water or riboflavin solution in the presence of Silwet L-77 (0.015%) for 4 h, and subsequently challenged with *Pst* DC3000. After indicated times, the leaves were collected. (**b**) Numbers of *Pst* DC3000 in leaves of WT and *npr1* mutant infected with *Pst* DC3000 inoculation. Each value is the mean ± SD of three replicates. dpi, days post inoculation.
