# Supplementary material for: Riboflavin-Induced Disease Resistance Requires the Mitogen-Activated Protein Kinases 3 and 6 in Arabidopsis thaliana
Source: PLoS One. 2016 Apr 7;11(4):e0153175. doi: 10.1371/journal.pone.0153175 (PMC4824526; doi:10.1371/journal.pone.0153175)
Supplement: S10 Fig — (DOCX) [file pone.0153175.s010.docx]

**
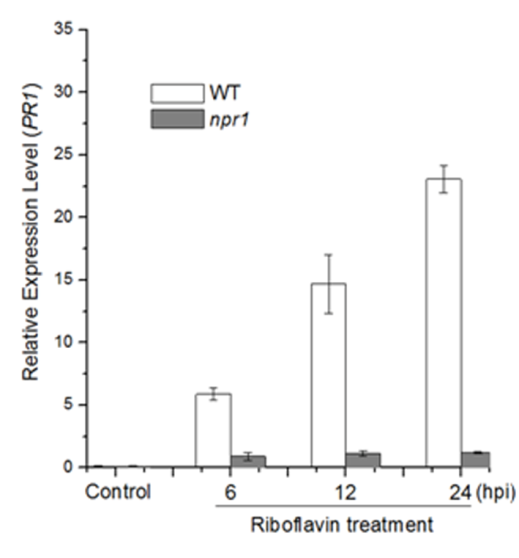
**

**S10 Fig.**

**S10 Fig. Effect of riboflavin on *PR1* gene expression in *npr1* mutant.** Real-time quantitative RT-PCR analyses showing induction of *PR1* gene expression in WT and *npr1* mutant plants upon infection with *Pst* DC3000 at 6, 12, and 24 hpi. The Arabidopsis ecotype Col-0 plants were sprayed with either water or riboflavin (0.6 mM) in the presence of Silwet L-77 (0.015%) and 4 hours later were challenged with *Pst* DC3000 for the different times. Arabidopsis *ACTIN2* was used as an internal control. Data are means ± SD of three replicates. hpi, hours post inoculation.
