# Supplementary material for: Riboflavin-Induced Disease Resistance Requires the Mitogen-Activated Protein Kinases 3 and 6 in Arabidopsis thaliana
Source: PLoS One. 2016 Apr 7;11(4):e0153175. doi: 10.1371/journal.pone.0153175 (PMC4824526; doi:10.1371/journal.pone.0153175)
Supplement: S11 Fig — (DOCX) [file pone.0153175.s011.docx]

**
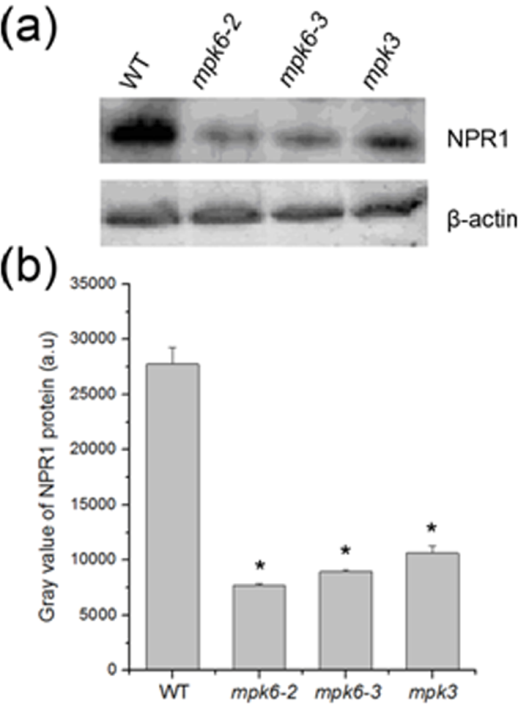
**

**S11 Fig.**

**S11 Fig.** **Effect of riboflavin on the expression of NPR1 protein in WT, *mpk3*, and *mpk6* mutant in response to *Pst* DC3000.** (**a**) Western blot with NPR1 antibody of protein extracts from WT and *mpk3* and *mpk6* mutant plants at 12 h with *Pst* DC3000 inoculation. (**b**) Quantitative analysis of the level of NPR1 in different Arabidopsis ecotype plants. Data represent the mean ± SD. Asterisks represent statistically significant difference to WT (student’s t-test: *p < 0.05)
