# Supplementary material for: Riboflavin-Induced Disease Resistance Requires the Mitogen-Activated Protein Kinases 3 and 6 in Arabidopsis thaliana
Source: PLoS One. 2016 Apr 7;11(4):e0153175. doi: 10.1371/journal.pone.0153175 (PMC4824526; doi:10.1371/journal.pone.0153175)
Supplement: S1 Table — (DOCX) [file pone.0153175.s012.docx]

**S1 Table. Primers for several genes.**

| Gene | Primer pairs (5’-3’) |
| --- | --- |
| *MPK3*  (At3g45640) | F: TGACGTTTGACCCCAACAGA |
|  | R: CTGTTCCTCATCCAGAGGCTG |
| *MPK6*  (At2g43790) | F: CCGACAGTGCATCCTTTAGCT |
|  | R: TGGGCCAATGCGTCTAAAAC |
| *PR1*  (At2g14610) | F: TCGGAGCTACGCAGAACAACT |
|  | R: TCTCGCTAACCCACATGTTCA |
| *LS*  (At2g44050) | F: TTGGAAGGAGCGATTGAGACT |
|  | R: CAAGATTTTGTGCAACAACACC |
| *RS*  (At2g22450) | F: TGAAAGGCGAAGACTTGGAGA |
|  | R: CGAGCTGAGACACCTGTGGAT |
| *ACTIN2*  (At3g18780) | F: GGCAAGTCATCACGATTGG |
|  | R: CAGCTTCCATTCCCACAAAC |

Note: F indicates forward and R indicates reverse.
